# Cigarette, e-cigarette and dual use among Malaysian adolescents: A secondary analysis of the 2017 and 2022 adolescent health surveys

**DOI:** 10.18332/tid/226883

**Published:** 2026-09-22

**Authors:** Kuang Kuay Lim, Maznieda Mahjom, S Maria Awaluddin, Noor Syaqilah Shawaluddin, Ying Ying Chan, Hamizatul Akmal Abd Hamid, Hui Li Lim, Kee Chee Cheong, Kuang Hock Lim

**Affiliations:** 1 Institute for Medical Research, National Institutes of Health, Ministry of Health Malaysia, Shah Alam, Selangor, Malaysia; 2 Institute for Public Health, National Institutes of Health, Ministry of Health Malaysia, Shah Alam, Selangor, Malaysia; 3 Institute for Clinical Research, National Institutes of Health, Ministry of Health Malaysia, Shah Alam, Selangor, Malaysia; 4 Biostatistics and Data Repository Sector, National Institutes of Health, Ministry of Health Malaysia, Shah Alam, Selangor, Malaysia; 5 Independent Researcher, Seri Kembangan, Selangor, Malaysia

**Keywords:** tobacco use, cigarette, e-cigarettes, dual use, Adolescent Health Survey (AHS)

## Abstract

**INTRODUCTION:**

Tobacco usage continues to pose a considerable public health challenge in Malaysia, with a predominant number of smokers initiating use during adolescence. The rapid increase of alternative nicotine products, especially e-cigarettes, has changed tobacco consumption trends among adolescents. This study examined changes in the prevalence of cigarette, e-cigarette, and dual use among Malaysian adolescents between 2017 and 2022.

**METHODS:**

This was a secondary analysis of cross-sectional data from the National Health and Morbidity Survey (NHMS) 2017 and 2022 Adolescent Health Survey (AHS), which employed a consistent multistage, stratified, cluster sampling design. Data were collected via self-administered questionnaires. Primary outcomes were past 30-day cigarette-only use, e-cigarette-only use, and dual use of both products. Weighted prevalence estimates and multivariable logistic regression analyses were applied to identify associated factors.

**RESULTS:**

The survey achieved response rates of 89.0% in both 2017 and 2022. Between 2017 and 2022, cigarette-only use declined from 4.9% to 0.9% (p<0.001), while e-cigarette-only use increased from 6.3% to 8.4% (p=0.002). Dual use decreased slightly from 5.6% to 4.5% (p=0.045). Multivariable logistic regression revealed that males, upper secondary students, and those with parental tobacco use had higher odds of tobacco use across all categories. Ethnic differences were also observed.

**CONCLUSIONS:**

The nicotine landscape among Malaysian adolescents has shifted significantly, with a sharp decline in cigarette smoking but a concerning rise in e-cigarette use. Males, upper secondary students, and those with parental tobacco use remain at higher risk. Future longitudinal studies should examine initiation patterns and the influence of peer factors, socioeconomic status, and regulatory changes.

## INTRODUCTION

In Malaysia, smoking remains a prevalent preventable cause of morbidity and mortality, exerting significant pressure on the healthcare system^1,2^. Despite continuous initiatives to regulate tobacco consumption, the prevalence of smoking among Malaysian adults has remained unchanged over the past decade^3^. This tendency is particularly concerning given that most individuals first smoke during their adolescence^4^, a critical phase in their developmental period when they can rapidly become dependent on nicotine, even with infrequent or recreational usage^5^. Initiating smoking at a young age significantly increases the likelihood of persisting the habit during adulthood^6^, thus necessitating that public health officials prioritize prevention efforts among adolescents.

The emergence of alternative nicotine products such as e-cigarettes and shisha has significantly altered the tobacco landscape in Malaysia^7^. These products are favored by youth due to their appealing flavors, aesthetic designs, and extensive marketing across both digital and physical retail environments^8,9^. E-cigarettes are frequently promoted as aids for adult smokers seeking to quit^10^. However, an increasing number of adolescents who have never smoked traditional cigarettes are experimenting with them^11^. Consequently, adolescents are changing their tobacco consumption habits. Rather than utilizing a singular substance, individuals are increasingly employing two or more nicotine products concurrently or in various ways^7,12^.

Adolescents who concurrently utilize multiple tobacco products face an elevated risk. Dual users generally have elevated nicotine dependence, reduced willingness to quit, and a greater tendency to partake in other hazardous activities^13^. Consuming a variety of items significantly elevates the overall exposure to nicotine and other detrimental substances^14^. Furthermore, dual use may represent a distinct behavioral pattern whereby adolescents combine products to increase nicotine intake rather than substituting one for the other^12^. In Malaysia and elsewhere, most tobacco control initiatives primarily focus on conventional cigarettes^15^. Current initiatives to curb smoking are ineffective due to regulatory issues in product development, including varying restrictions regarding advertising, flavoring, and nicotine concentrations^15,16^.

The early initiation of tobacco use is a significant concern in Malaysia. The Tobacco and E-Cigarette Survey among Malaysian Adolescents (TECMA) 2016 revealed that 11.7% of students aged 10–19 years were current smokers, with 78.7% initiating smoking before the age of 14 years^17^. The National Health and Morbidity Survey (NHMS) 2022: Adolescent Health Survey (AHS) indicated a significant increase in e-cigarette usage, with 14.9% of adolescents aged 13–17 years partaking in this activity^7^. Prolonged exposure to marketing and product promotions, whether digital or physical, increases the likelihood of adolescents experimenting with new products, particularly among non-smokers. Nicotine consumption in teenagers can impede critical brain development. The brain processes governing impulse control, decision-making, and emotional regulation are still maturing during this phase, rendering adolescents more susceptible to reliance on these systems and prone to maladaptive behaviour^18,19^.

The Control of Smoking Products for Public Health Act 2024 (Act 852) is enhancing Malaysia’s regulations by imposing stricter measures on nicotine products^20^. Nonetheless, issues with enforcement among youngsters and the persistent promotion of vape liquids indicate a necessity for methods that are both comprehensive and flexible. Although scarce Malaysian data suggest that dual/poly tobacco use is concerning, prior research, such as TECMA 2016 (5.2% dual/poly use), may not adequately reflect current behaviors due to the swift growth of the vaping business and evolving accessibility^17^. Thus, this study employed a cross-sectional design to examine changes in prevalence between the two survey waves. The study aimed to examine changes in the population prevalence of cigarette-only, e-cigarette-only, and dual use among Malaysian adolescents by analyzing secondary data from two cross-sectional surveys conducted in 2017 and 2022, and to identify associated sociodemographic factors.

## METHODS

### Study design and data source

This study involved a secondary analysis of data from the National Health and Morbidity Survey (NHMS): Adolescent Health Survey (AHS), a nationally representative, school-based survey designed to monitor health-risk behaviors among Malaysian secondary school students. Data from two survey waves, conducted in 2017^20^ and 2022^7^, were analyzed to compare prevalence estimates and associated factors between the two survey waves. Both cycles employed an identical cross-sectional design, ensuring comparability of estimates across time.

### Sampling strategy and population

The survey employed multistage, stratified cluster sampling to obtain a representative sample of students from all states and federal territories in Malaysia. This comprehensive sampling approach accounted for the country’s geographical and sociodemographic diversity, thereby enabling generalization of the findings to the broader adolescent population nationwide. For both the 2017 and 2022 surveys^7,20^, the Ministry of Education Malaysia supplied the most recent official school enrolment sampling frames, namely the 2016 frame for NHMS: AHS 2017 and the 2021 frame for NHMS: AHS 2022. The sampling process commenced with the stratification of the national framework by state, ensuring proportional representation across all regions. In the first stage of sampling, the primary sampling units (PSUs) were defined as schools. Schools were selected from each stratum using probability proportional to enrolment size (PPS). In the second stage, within each selected school, classes were randomly chosen as secondary sampling units (SSUs). All students in the selected classes were invited to participate in the survey.

### Inclusion criteria

The study included secondary school students from public, government-aided schools, and private schools in Malaysia. Students were eligible if they were enrolled in Forms 1 to 5 (aged approximately 13–17 years). Students with special needs or learning disabilities that prevented them from completing the self-administered questionnaire independently were excluded.

### Survey instrument and measures

Data were collected using a standardized, self-administered, anonymous questionnaire developed through a rigorous process, including a comprehensive literature review, the adoption of validated items from previous Global School-based Student Health Surveys (GSHS) conducted in Malaysia^21^, and input from a panel of local experts in adolescent health, tobacco control, and survey methodology. It was pre-tested and piloted in a sample of students not included in the final survey to assess question comprehension, clarity, reliability, and the average time required for completion. The questionnaire was organized into distinct sections covering a broad range of topics, including demographic characteristics, substance use, dietary behaviors, and mental health. For the present analysis, the following variables were extracted and examined.

*Dependent variables*

Tobacco-related outcomes were defined as self-reported use within the 30 days preceding the survey. Three dependent variables were examined. First, current cigarette use was assessed as a binary variable (yes, no) based on the question: ‘In the past 30 days, on how many days did you smoke cigarettes?’. Respondents reporting smoking on one or more days were classified as current cigarette users. Second, current e-cigarette or vape use was assessed as a binary variable (yes, no) based on the question: ‘In the past 30 days, on how many days did you use an e-cigarette or vape?’. Respondents reporting use on one or more days were classified as current e-cigarette users. Third, dual use was derived as a binary variable (yes, no) by combining the two preceding measures. Students reporting both current cigarette use and current e-cigarette use were classified as dual users. Fourth, single use was defined as the use of only one type of tobacco product (either cigarettes only or e-cigarettes only) within the past 30 days.

*Independent variables*

The sociodemographic independent variables were selected a priori based on existing literature on adolescent tobacco use^7,17^. These variables included sex (male, female); education level grouped as lower secondary (Forms 1–3) or upper secondary (Forms 4–5); ethnicity (Malay, Chinese, Indian, Bumiputera Sabah, Bumiputera Sarawak, or other); parental marital status (married including living together, divorced/separated/widowed); and parental tobacco use (yes, no) based on the student’s perception of whether either parent currently uses any tobacco products.

### Data collection procedure and quality assurance

Data were collected through a standardized procedure designed to ensure accuracy, privacy, and confidentiality. Written informed consent was obtained from parents or guardians, and passive assent was obtained from students on the day of the survey. Participation was voluntary, anonymous, and confidential, with students free to decline or withdraw at any time. Trained data collectors administered the survey in classroom settings during a designated school period. Teachers were instructed not to observe student responses to minimize social desirability bias. Standardized instructions were read aloud to the entire group to ensure consistency. Although the questionnaire was self-administered, data collectors provided support for students with lower literacy, helping them read and comprehend items. They also clarified questions as needed without influencing responses and addressed any technical issues with the answer sheets. Upon completion, students placed their questionnaires into sealed envelopes to reinforce anonymity.

### Ethical considerations

Ethical approval for both surveys was obtained from the Medical Research and Ethics Committee (MREC), Ministry of Health Malaysia, for the 2017 survey (NMRR-16-698-30042) and the 2022 survey (NMRR-21-157-58261). Permission was also granted by the Ministry of Education, Malaysia, and the respective school principals. Participation was voluntary, and written informed consent was obtained from both students and their parents or guardians.

### Statistical analysis

The raw data underwent cleaning and validation, including checks for logical inconsistencies, outliers, and missing values. Missing data for the dependent variables were minimal (<5%) and excluded from the analysis. For independent variables, listwise deletion was applied in the multivariable regression models. To ensure representativeness, data were weighted using a three-step procedure: 1) design weights based on selection probabilities at school and class levels; 2) non-response adjustments; and 3) post-stratification calibration to match the national population distribution by age, sex, and ethnicity. All analyses were performed using the Complex Samples module in IBM SPSS Statistics Version 22. Descriptive statistics were first computed to report the weighted prevalence of the dependent variables with 95% confidence intervals (CIs). Bivariate associations between independent variables and tobacco use outcomes were then examined using Rao-Scott chi-squared tests. Finally, multivariable logistic regression models were constructed to identify factors associated with single and dual tobacco use, adjusting for sex, education level, ethnicity, parental tobacco use, and parental marital status. Multicollinearity was assessed using variance inflation factors (VIF <10). Separate models were constructed for each survey year (2017 and 2022) and for each outcome (single and dual use). Variables with a bivariate p≤0.25 were included in the initial models to avoid excluding potentially important variables. Results are presented as adjusted odds ratios (AORs) with 95% CIs.

## RESULTS

### Sample characteristics

A total of 27497 students participated in 2017 and 33523 in 2022, with a response rate of 89.0% for both surveys. Males comprised 49.6% of the sample in 2017 and 50.0% in 2022. Parental tobacco use was reported by 43.6% of students in 2017 and 43.4% in 2022. Ethnic composition was Malay (2017: 63.1% vs 2022: 63.0%), Chinese (16.7% vs 18.1%), Indian (7.0% vs 6.0%), Bumiputera Sabah (7.0% vs 5.6%), Bumiputera Sarawak (4.5% vs 5.1%), and Other (1.8% vs 2.2%). Lower secondary students comprised 61.0% in 2017 and 62.8% in 2022. Married parents accounted for 87.4% in 2017 and 85.1% in 2022 (Table 1).

**Table 1:** Sociodemographic characteristics of Malaysian secondary school adolescents from the nationally representative, school-based National Health and Morbidity Survey (NHMS): Adolescent Health Survey (AHS) in 2017 (N=27497) and 2022 (N=33523)

*Characteristics*	*2017* *n (%)*	*2022* *n (%)*
**Sex**		
Male	13135 (49.6)	15493 (50.0)
Female	14362 (50.4)	18030 (50.0)
**Parental tobacco use**		
No	14510 (56.4)	17956 (56.6)
Yes	11083 (43.6)	13642 (43.4)
**Ethnicity**		
Malay	18713 (63.1)	23125 (63.0)
Chinese	4100 (16.7)	5085 (18.1)
Indian	1428 (7.0)	1556 (6.0)
Bumiputera Sabah	1781 (7.0)	1722 (5.6)
Bumiputera Sarawak	921 (4.5)	1241 (5.1)
Other	554 (1.8)	794 (2.2)
**Educational level**		
Lower secondary	17042 (61.0)	20578 (62.8)
Upper Secondary	10455 (39.0)	12945 (37.2)
**Parent marital status**		
Married	23544 (87.4)	28070 (85.1)
Divorced/separated/widowed	3387 (12.6)	4844 (14.9)

### Prevalence of tobacco use (2017 vs 2022)

The proportion of non-users increased significantly from 83.2% (95% CI: 82.1–84.2) to 86.2% (95% CI: 85.1–87.3), while cigarette-only use decreased significantly from 4.9% (95% CI: 4.5–5.4) to 0.9% (95% CI: 0.8–1.0). E-cigarette-only use also rose significantly from 6.3% (95% CI: 5.9–6.7) to 8.4% (95% CI: 7.7–9.1); however, dual use declined from 5.6% (95% CI: 5.1–6.2) to 4.5% (95% CI: 3.9–5.1) (Table 2).

**Table 2:** Prevalence of tobacco product use among adolescents in Malaysia from the NHMS: AHS in 2017 (N=27497) and 2022 (N=33523)

*Tobacco product use*	*2017* *% (95% CI)*	*2022* *% (95% CI)*	p*
**Non-use**	83.2 (82.1–84.2)	86.2 (85.1–87.3)	**<0.001**
**Cigarettes only**	4.9 (4.5–5.4)	0.9 (0.8–1.0)	**<0.001**
**E-cigarettes only**	6.3 (5.9–6.7)	8.4 (7.7–9.1)	**0.002**
**Dual use**	5.6 (5.1–6.2)	4.5 (3.9– 5.1)	**0.045**

Tobacco product use is defined as self-reported use within the 30 days preceding the survey.

* Values from chi-squared test, significance at p<0.05.

### Prevalence of tobacco use by sociodemographic characteristic

Significant sex differences were found, with males exceeding females in single use (11.5% vs 1.4% in 2017; 14.3% vs 4.3% in 2022) and dual use (16.2% vs 5.4% in 2017; 7.5% vs 1.5% in 2022). School level also showed significant differences, with upper secondary students surpassing lower secondary students in single use (6.8% vs 6.1% in 2017; 11.3% vs 8.1% in 2022) and dual use (11.6% vs 10.3% in 2017; 6.0% vs 3.5% in 2022). Dual use prevalence varied significantly by ethnicity, highest among Bumiputera Sarawak (14.7% in 2017; 9.1% in 2022) and lowest among Chinese students (5.7% in 2017; 1.9% in 2022). Parental tobacco use was significantly associated with higher prevalence of single use (8.4% vs 3.9% in 2017; 11.3% vs 7.4% in 2022) and dual use (11.9% vs 9.3% in 2017; 6.0% vs 3.0% in 2022). Finally, family structure significantly impacted prevalence, with divorced/separated/widowed showing higher prevalence rates than married households for single use (8.3% vs 6.0% in 2017; 12.1% vs 8.8% in 2022) and dual use (12.9% vs 10.3% in 2017; 6.7% vs 4.0% in 2022) (Table 3).

**Table 3:** Comparison of single and dual tobacco product use prevalence, by sociodemographic characteristics, among adolescents in Malaysia from the NHMS: AHS in 2017 (N=27497) and 2022 (N=33523)

*Variables*	*Category*	*Year*	*Non-use* *% (95% CI)*	*Dual use* *% (95% CI)*	*Single use* *% (95% CI)*	*Total*	p*
**Sex**	Male	2017	72.2 (70.4–74.0)	16.2 (15.2–17.3)	11.5 (10.4–12.7)	13122	<0.001
		2022	78.2 (76.3–80.0)	7.5 (6.5–8.6)	14.3 (13.2–15.5)	15395	<0.001
	Female	2017	93.2 (92.4–93.9)	5.4 (4.8–6.0)	1.4 (1.1–1.8)	14351	<0.001
		2022	94.3 (93.7–94.7)	1.5 (1.2–1.8)	4.3 (3.8–4.7)	17962	<0.001
**Education level**	Lower secondary	2017	83.6 (82.2–84.9)	10.3 (9.5–11.1)	6.1 (5.3–7.1)	17027	0.144
		2022	88.3 (87.3–89.3)	3.5 (3.1–4.0)	8.1 (7.3–9.0)	20445	<0.001
	Upper secondary	2017	81.6 (79.7–83.3)	11.6 (10.4–12.8)	6.8 (5.9–8.0)	10446	0.144
		2022	82.7 (80.5–84.7)	6.0 (5.0–7.2)	11.3 (10.0–12.6)	12912	<0.001
**Ethnicity**	Malay	2017	82.1 (80.9–83.3)	11.4 (10.7–12.2)	6.4 (5.8–7.2)	18698	<0.001
		2022	84.4 (83.0–85.8)	4.9 (4.3–5.6)	10.7 (9.8–11.6)	23013	<0.001
	Chinese	2017	91.4 (89.6–93.0)	5.7 (4.7–7.0)	2.9 (2.1–3.8)	4096	<0.001
		2022	95.0 (93.9–95.9)	1.9 (1.4–2.5)	3.1 (2.5–4.0)	5064	<0.001
	Indian	2017	82.1 (76.6–86.5)	9.8 (8.1–12.0)	8.0 (4.6–13.6)	1427	<0.001
		2022	91.0 (88.4–93.1)	2.1 (1.4–3.2)	6.8 (5.0–9.3)	1548	<0.001
	Bumiputera Sabah	2017	76.0 (71.6–79.9)	14.5 (12.3–17.0)	9.5 (7.1–12.6)	1780	<0.001
		2022	83.1 (79.8–85.9)	5.5 (4.0–7.5)	11.4 (9.7–13.4)	1710	<0.001
	Bumiputera Sarawak	2017	73.9 (68.5–78.7)	14.7 (11.6–18.6)	11.3 (8.6–14.8)	918	<0.001
		2022	78.3 (74.2–81.9)	9.1 (6.6–12.5)	12.6 (10.7–14.7)	1239	<0.001
	Other	2017	77.8 (71.7–83.0)	13.9 (10.2–18.8)	8.2 (5.2–12.8)	554	<0.001
		2022	79.2 (75.1–82.8)	6.3 (4.0–9.7)	14.5 (11.8–17.8)	783	<0.001
**Parental tobacco use**	No	2017	86.8 (85.7–87.7)	9.3 (8.5–10.1)	3.9 (3.4–4.5)	14508	<0.001
		2022	89.6 (88.5–90.6)	3.0 (2.6–3.5)	7.4 (6.7–8.1)	17952	<0.001
	Yes	2017	79.6 (78.1–81.0)	11.9 (11.1-12.8)	8.4 (7.4–9.6)	11076	<0.001
		2022	82.7 (81.2-84.0)	6.0 (5.2-6.9)	11.3 (10.4–12.3)	13630	<0.001
**Parental marital status**	Married	2017	83.7 (82.6–84.7)	10.3 (9.6–11.0)	6.0 (5.4–6.7)	23525	<0.001
		2022	87.2 (86.0–88.2)	4.0 (3.6–4.6)	8.8 (8.1–9.6)	27939	<0.001
	Divorced/separated/widowed	2017	78.8 (76.7–80.8)	12.9 (11.4–14.6)	8.3 (6.9–9.8)	3382	<0.001
		2022	81.2 (79.2–83.0)	6.7 (5.6–8.0)	12.1 (10.8–13.5)	4819	<0.001

Tobacco product use is defined as self-reported use within the 30 days preceding the survey. Single use: use of only one product (cigarette only or e-cigarette-only). Dual use: use of both products.

* Values from chi-squared test, significance at p<0.05.

### Factors associated with tobacco use

Multivariable logistic regression showed that males had significantly greater odds than females for both single use (2017: AOR=4.25; 95% CI: 3.69–4.91; 2022: AOR=4.74; 95% CI: 4.20–5.34) and dual use (2017: AOR=12.86; 95% CI: 9.71–17.03; 2022: AOR=6.76; 95% CI: 5.22–8.75). Upper secondary students also had significantly higher odds than lower secondary students for single use (2017: AOR=1.25; 95% CI: 1.08–1.45; 2022: AOR=1.60; 95% CI: 1.35–1.89) and dual use (2017: AOR=1.40; 95% CI: 1.09–1.81; 2022: AOR=1.93; 95% CI: 1.57–2.38). Parental tobacco use was significantly associated with higher odds of single use (2017: AOR=1.46; 95% CI: 1.31–1.63; 2022: AOR=1.65; 95% CI: 1.48–1.84) and dual use (2017: AOR=2.59; 95% CI: 2.17–3.09; 2022: AOR=2.13; 95% CI: 1.82–2.49). Adolescents from divorced/separated/widowed households had significantly higher odds than those from married households for single use (2017: AOR=1.44; 95% CI: 1.23–1.69; 2022: AOR=1.54; 95% CI: 1.35–1.77) and dual use (2017: AOR=1.64; 95% CI: 1.33–2.03; 2022: AOR=1.94; 95% CI: 1.61–2.33). Compared to Chinese students, all other ethnic groups had significantly higher odds of single use in both years, as well as dual use in both years, except for Indians in 2022, where the difference was not significant (AOR=1.29; 95% CI: 0.75–2.22) (Table 4).

**Table 4:** Adjusted odds ratios (AORs) and 95% confidence intervals (CIs) for factors associated with single and dual tobacco product use among adolescents in Malaysia from the NHMS: AHS in 2017 (N=27497) and 2022 (N=33523), and the observed trend from 2017 to 2022

*Predictor*	*Comparison*	*2017*	*2022*	*2017*	*2022*	*Trend* *2017–2022*
		*Single use* *AOR (95% CI)*	*Single use* *AOR (95% CI)*	*Dual use* *AOR (95% CI)*	*Dual use* *AOR (95% CI)*	
**Sex**	Male vs Female	4.25 (3.69–4.91)	4.74 (4.20–5.34)	12.86 (9.71–17.03)	6.76 (5.22–8.75)	Single ↑ Dual ↓
**Education level**	Upper vs Lower Secondary	1.25 (1.08–1.45)	1.60 (1.35–1.89)	1.40 (1.09–1.81)	1.93 (1.57–2.38)	Single ↑ Dual ↑
**Parental tobacco use**	Yes vs No	1.46 (1.31–1.63)	1.65 (1.48–1.84)	2.59 (2.17–3.09)	2.13 (1.82–2.49)	Single ↑ Dual ↓
**Parental marital status**	Divorced/separated/widowed vs Married	1.44 (1.23–1.69)	1.54 (1.35–1.77)	1.64 (1.33–2.03)	1.94 (1.61–2.33)	Single ↑ Dual ↑
**Ethnicity**	Malays	2.44 (1.90–3.13)	3.84 (2.87–5.14)	2.80 (1.95–4.01)	2.91 (2.08–4.07)	Single ↑ Dual ↓
	Indians	2.12 (1.45–3.11)	2.66 (1.63–4.33)	4.25 (1.92–9.42)	1.29 (0.75–2.22)	Single ↑ Dual ↓
	Bumiputera Sabah	3.53 (2.59–4.80)	4.38 (3.05–6.29)	4.37 (2.66–7.20)	3.39 (2.14–5.36)	Single ↑ Dual ↓
	Bumiputera Sarawak	3.54 (2.44–5.13)	4.70 (3.33–6.64)	5.94 (3.69–9.57)	5.86 (3.61–9.50)	Single ↑ Dual →
	Other	3.30 (2.17–5.00)	6.39 (4.22–9.68)	4.34 (2.40–7.86)	4.40 (2.41–8.01)	Single ↑ Dual ↓

Tobacco product use is defined as self-reported use within the 30 days preceding the survey. Reference category for ethnicity: Chinese. Trend key: ↑ increased risk, ↓ decreased risk, → stable risk. AOR: adjusted odds ratio. CI: confidence interval.

## DISCUSSION

This study adds to the existing literature by providing a nationally representative comparison of cigarette, e-cigarette, and dual use among Malaysian adolescents using data from the 2017 and 2022 Adolescent Health Survey (AHS). A notable decline in exclusive cigarette consumption, minor fluctuations in dual usage, and an increase in exclusive e-cigarette consumption highlight the changing patterns of adolescent nicotine consumption. Education level was a significant factor, with upper secondary students exhibiting a higher prevalence of tobacco use compared to lower secondary students.

The significant reduction of exclusive cigarette smoking from 4.9% in 2017 to 0.9% in 2022 may be attributed to conventional tobacco control strategies in Malaysia^7^. Measures such as school-based health education, age restrictions on sales, taxation laws, and mass media campaigns are likely factors that have contributed to the decline in cigarette smoking among teenagers^22,23^. Similarly, countries such as the United States, Canada, and some European nations have reported declines in teen smoking rates, which coincide with stronger laws and public health messaging^24,25^. These associations underscore the importance of maintaining evidence-based approaches to reduce conventional cigarette use.

In contrast, the proportion of adolescents using only e-cigarettes increased from 6.3% to 8.4% during the same period. This trend is supported by reports indicating that e-cigarette use among youth is rising even as traditional cigarette use declines^26^. The increase is attributed to perceptions of reduced harm, the appeal of technological innovation, the availability of flavored products, and greater social acceptance^27-29^. In addition, before the enactment of the Control of Smoking Products for Public Health Act (Act 852) in 2024^30^, the regulatory environment may have facilitated adolescents’ access to vaping devices, particularly through online or informal channels. These findings indicate that, although public health measures have reduced traditional smoking, they have not fully addressed the challenges posed by emerging nicotine delivery technologies^28,29^.

Concurrent use of cigarettes and e-cigarettes presents a significant public health concern. Although the prevalence of dual use among adolescents is declining slightly, those who engage in this behavior remain at increased risk of nicotine addiction and regular smoking^31^. Patterns of dual use illustrate the complexity of adolescent nicotine consumption, indicating that some adolescents may combine both products to increase nicotine intake rather than substituting one for the other. This observation is consistent with studies from Europe and North America, which report that dual users engage more frequently in higher risk behaviors and exhibit lower levels of protective behavior than exclusive users^31,32^.

Education level was significantly associated with nicotine use, with upper secondary students exhibiting higher prevalence of cigarette, e-cigarette, and dual use compared to lower secondary students. This finding aligns with research demonstrating that upper secondary students have higher rates of initiation and frequency of nicotine use, likely due to greater autonomy, peer influence, and exposure to adult behaviours^7,32^. Early use among lower secondary students is associated with a higher risk of nicotine addiction and long-term health consequences. Factors contributing to early initiation include the appeal of flavored or discreet devices, ease of access, and peer dynamics within adolescent groups^7,33,34^.

Gender differences also persist in adolescent nicotine use. Male adolescents exhibit higher rates of cigarette, e-cigarette, and dual use compared to females, a pattern observed in local^7,17^ and international studies^2,5^. This disparity is often linked to social norms that associate masculinity with risk-taking and smoking behaviors. Male adolescents are more likely to participate in peer groups where nicotine use is prevalent, which encourages experimentation and regular use. In contrast, female adolescents, despite a modest increase in e-cigarette use, are less likely to initiate use due to heightened social stigma and stricter supervision^8,9^. These patterns highlight the need for gender-specific interventions that address the distinct motivations and social influences affecting boys and girls.

Parental tobacco use was associated with higher odds of adolescent tobacco use, consistent with its role as a behavioral model and potential indicator of permissive attitudes toward smoking. Globally, evidence indicates that parental tobacco use is a primary risk factor for adolescent tobacco and nicotine consumption, as adolescents exposed to parental tobacco use are more likely to initiate use and persist in using both traditional and electronic cigarettes^24,25^.

The trends observed in Malaysia correspond with global findings concerning prevalence and risk factor patterns. Cross-national studies of adolescents aged 12–16 years across multiple countries have shown a rise in e-cigarette consumption alongside a decline in traditional cigarette use, while dual usage has intensified in various contexts^35^. Studies in Korea and Europe identified male gender, older age, and parental tobacco use as significant predictors of adolescent nicotine consumption^34^. The recognized risk factors in Malaysia are not exclusive; they reflect broader social and behavioral determinants common across diverse cultural settings. Longitudinal cohort studies demonstrate that dual users and e-cigarette users are at a higher risk of developing nicotine dependence and future regular cigarette use^12,14^. Although this study did not directly assess dependence, the finding that dual use is more common among adolescents with smoking parents raises concerns about a pathway toward dependence and poor long-term health outcomes. Cultural norms and social acceptance are likely to influence adolescent behavior in Malaysia. Male adolescents may receive encouragement to take risks, whereas female adolescents often face greater criticism from family and peers. The interplay of education level, gender, and parental factors demonstrates the complex dynamics of adolescent nicotine use, where individual characteristics interact with familial and social factors^22^.

### Limitations

This study has several limitations. First, the cross-sectional design inhibits causal inference regarding the relationship between parental tobacco use and adolescent nicotine consumption. Second, relying on self-reported tobacco and e-cigarette use may be affected by recall bias or social desirability bias, and the lack of biochemical validation (e.g. cotinine levels) to confirm smoking status is a further limitation. Third, parental tobacco use was based on adolescent perception rather than direct parental report, which may introduce misclassification bias. Fourth, the study did not account for several potential confounders, including peer influence, household income, parental supervision, academic achievement, and perceptions of harm, which may have contributed to residual confounding. Finally, the lack of information on the frequency, intensity, and nicotine concentration of e-cigarette use limited the assessment of dependence and exposure risk. Nevertheless, the study also has important strengths. The large, nationally representative samples from the NHMS provide robust estimates that are generalizable to Malaysian adolescents. The use of standardized questionnaires and consistent data collection methods across both survey years enables valid comparisons over time. Furthermore, the comprehensive collection of sociodemographic and behavioral variables enabled multivariable adjustment, thereby enhancing the reliability of the observed associations.

## CONCLUSIONS

While cigarette smoking among Malaysian adolescents has declined, the rise of e-cigarette and dual use signals a shift in nicotine consumption patterns. Male adolescents, upper secondary students, and those with smoking parents remain at higher risk, underscoring the influence of demographic and familial factors. The observed decline in cigarette use alongside the rise in e-cigarette use suggests that while existing tobacco control efforts may have contributed to reducing conventional smoking, emerging nicotine products require comprehensive, targeted strategies. Interventions should address the higher risk observed among males, upper secondary students, and those with smoking parents, and include parental involvement and enhanced education. Future research should prioritize longitudinal studies on initiation, progression, and cessation, incorporating risk factors such as peer influence, parental oversight, socioeconomic status, and risk perceptions.

## Data Availability

The data supporting this research are available from the authors on reasonable request.
